# 1-(2,4-Dinitro­phen­yl)-2-(1,2,3,4-tetra­hydro­naphthalen-1-yl­idene)hydrazine

**DOI:** 10.1107/S1600536810028680

**Published:** 2010-07-24

**Authors:** M. Danish, Masood Hamid, M. Nawaz Tahir, Nazir Ahmad, Sabiha Ghafoor

**Affiliations:** a Department of Chemistry, University of Sargodha, Sargodha, Pakistan; b Department of Physics, University of Sargodha, Sargodha, Pakistan

## Abstract

In the title compound, C_14_H_14_N_4_O_4_, the dihedral angle between the benzene rings is 10.42 (8)°. The nitro groups make dihedral angles of 5.3 (2) and 6.47 (15)° with their parent ring and are oriented at 11.2 (3)° with respect to each other. An intra­molecular N—H⋯O hydrogen bond completes an *S*(6) ring motif. In the crystal, mol­ecules are linked by C—H⋯O inter­actions, thus forming (010) chains in which *R*_2_^2^(13) ring motifs are present. There also exist aromatic π–π stacking inter­actions [centroid–centroid separation = 3.7046 (9) Å].

## Related literature

For a related structure, see: Girgisa *et al.* (2003 ▶). For graph-set notation, see: Bernstein *et al.* (1995 ▶).
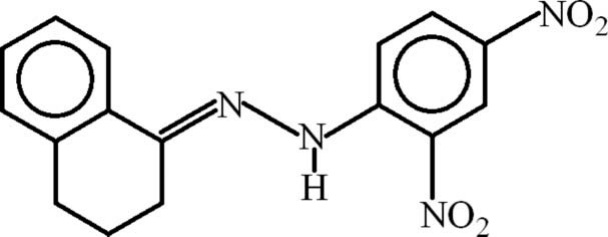


## Experimental

### 

#### Crystal data

C_16_H_14_N_4_O_4_
*M* *_r_* = 326.31
Monoclinic, 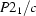

*a* = 14.8627 (8) Å
*b* = 13.8704 (7) Å
*c* = 7.3493 (4) Å
β = 99.211 (3)°
*V* = 1495.53 (14) Å^3^
*Z* = 4
Mo *K*α radiation
μ = 0.11 mm^−1^
*T* = 296 K
0.34 × 0.25 × 0.22 mm

#### Data collection

Bruker Kappa APEXII CCD diffractometer
Absorption correction: multi-scan (*SADABS*; Bruker, 2005 ▶) *T*_min_ = 0.966, *T*_max_ = 0.975
13873 measured reflections
3684 independent reflections
2275 reflections with *I* > 2σ(*I*)
*R*_int_ = 0.036

#### Refinement

*R*[*F* ^2^ > 2σ(*F* ^2^)] = 0.052
*wR*(*F* ^2^) = 0.143
*S* = 1.03
3684 reflections
217 parameters
H-atom parameters constrained
Δρ_max_ = 0.38 e Å^−3^
Δρ_min_ = −0.19 e Å^−3^

### 

Data collection: *APEX2* (Bruker, 2009 ▶); cell refinement: *SAINT* (Bruker, 2009 ▶); data reduction: *SAINT*; program(s) used to solve structure: *SHELXS97* (Sheldrick, 2008 ▶); program(s) used to refine structure: *SHELXL97* (Sheldrick, 2008 ▶); molecular graphics: *ORTEP-3* (Farrugia, 1997 ▶) and *PLATON* (Spek, 2009 ▶); software used to prepare material for publication: *WinGX* (Farrugia, 1999 ▶) and *PLATON*.

## Supplementary Material

Crystal structure: contains datablocks global, I. DOI: 10.1107/S1600536810028680/hb5560sup1.cif

Structure factors: contains datablocks I. DOI: 10.1107/S1600536810028680/hb5560Isup2.hkl

Additional supplementary materials:  crystallographic information; 3D view; checkCIF report

## Figures and Tables

**Table 1:** Hydrogen-bond geometry (Å, °)

*D*—H⋯*A*	*D*—H	H⋯*A*	*D*⋯*A*	*D*—H⋯*A*
N2—H2*A*⋯O1	0.86	1.99	2.5976 (19)	127
C2—H2⋯O2^i^	0.93	2.57	3.426 (2)	153
C15—H15⋯O1^i^	0.93	2.52	3.235 (2)	134

Symmetry code: (i) 
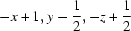
.

**Fig. 1.**
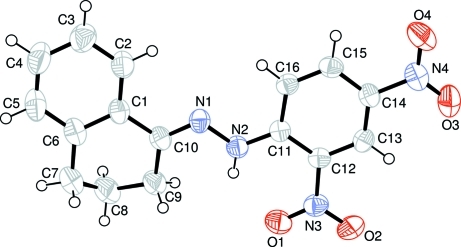
View of (I) with displacement ellipsoids drawn at the 50% probability level.

**Fig. 2.**
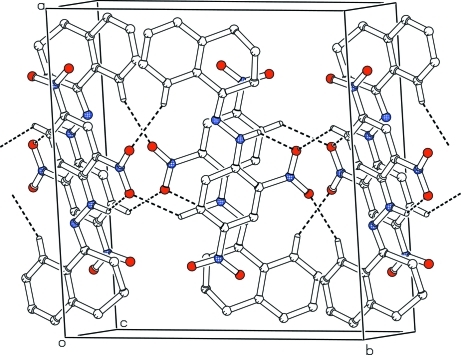
The partial packing of (I), which shows that molecules form polymeric chains extending along b axis.

## supplementary crystallographic information

### Comment

The title compound (I, Fig. 1) has been prepared for the chlorination and bromonition. Various properties of (I) as well as their derivatives will be undertaken.

The crystal structures of (II) *i.e.*, 6-amino-4-(4-chlorophenyl)-1,2-dihydro-1-[(2,3-dihydroindene- 1(1*H*)-ylidene)amino]-2-oxo-3,5-pyridinedicarbonitrile (Girgisa *et al.*, 2003) has been published which contain 3,4-dihydronaphthalen-1(2*H*)-ylidene moiety which is also present in (I). The other annilinic group i.e, (2,4-dinitrophenyl)hydrazine is very common.

In (I), the group A (C1—C7/C10) of 3,4-dihydronaphthalen-1(2*H*)-ylidene moiety is planar with r. m. s. deviation of 0.0051 Å. The C-atoms, C8 and C9 are at a distance of 0.8224 (33) and 0.2986 (27) Å from the mean square plane of A. Similarly nitrogen atom N1 is at a distance of -0.2603 (24) Å from the same. The phenyl ring B (C11—C16) is planar with r. m. s. deviation of 0.0053 Å and N2 is at 0.0189 (23) Å from it. The dihedral angle between A/B is 10.51 (6)°. The nitro groups C (O1/N3/O2) and D (O3/N4/O4) are of course planar. The dihedral angle between B/C, B/D and C/D is 5.26 (24), 6.47 (15) and 11.17 (25)°, respectively. There exist an intramolecular H-bonding of N—H···O type completing an S(6) (Fig. 2) ring motif (Bernstein *et al.*, 1995). The molecules are stabilized in the form of infinite one dimensional polymeric chains due to C—H···O type of intermolecular H-bondings (Table 1, Fig. 2) extending along the *b* axis and in these chains *R*_2_^2^(13) ring motifs are present. There exist π—π interaction between the centroids of phenyl rings of annilinic group at a distance of 3.7046 (9) Å [symmetry: 1 - *x*, - *y*, - *z*]. The π-interaction is present (Table 1) between the nitro group not involved in the intramolecular H-bonding and the phenyl ring of (2,4-dinitrophenyl)hydrazine moiety.

### Experimental

2,4-Dinitrophenylhydrazine (1.518 g, 7.67 mmol) was added to 50 ml of distilled methanol with constant stirring at room temperature in a 100-ml round bottom flask. Then 1-tetralon 1 ml (1.098 g, 7.5 mmol) was added to it and 3–4 drops of conc. HCl were also added into the reaction mixture. The mixture was refluxed for 4 h, and then brought to room temperature. A dark red solid was obtained which purified by repeated crystallization from chloroform to obtain dark red prisms of (I). These crystals had sharp 532.6 K melting point.

### Refinement

The H-atoms were positioned geometrically (N—H = 0.86, C–H = 0.93–0.97 Å) and refined as riding with *U*_iso_(H) = x*U*_eq_(C, N), where x = 1.2 for all H-atoms.

### Crystal data

**Table:**

C_16_H_14_N_4_O_4_	*F*(000) = 680
*M**_r_* = 326.31	*D*_x_ = 1.449 Mg m^−^^3^
Monoclinic, *P*2_1_/*c*	Mo *K*α radiation, λ = 0.71073 Å
Hall symbol: -P 2ybc	Cell parameters from 2225 reflections
*a* = 14.8627 (8) Å	θ = 2.1–25.2°
*b* = 13.8704 (7) Å	µ = 0.11 mm^−^^1^
*c* = 7.3493 (4) Å	*T* = 296 K
β = 99.211 (3)°	Prism, dark red
*V* = 1495.53 (14) Å^3^	0.34 × 0.25 × 0.22 mm
*Z* = 4	

### Data collection

**Table:**

Bruker Kappa APEXII CCD diffractometer	3684 independent reflections
Radiation source: fine-focus sealed tube	2275 reflections with *I* > 2σ(*I*)
graphite	*R*_int_ = 0.036
Detector resolution: 7.50 pixels mm^-1^	θ_max_ = 28.3°, θ_min_ = 2.8°
ω scans	*h* = −19→18
Absorption correction: multi-scan (*SADABS*; Bruker, 2005)	*k* = −18→18
*T*_min_ = 0.966, *T*_max_ = 0.975	*l* = −9→9
13873 measured reflections	

### Refinement

**Table:**

Refinement on *F*^2^	Primary atom site location: structure-invariant direct methods
Least-squares matrix: full	Secondary atom site location: difference Fourier map
*R*[*F*^2^ > 2σ(*F*^2^)] = 0.052	Hydrogen site location: inferred from neighbouring sites
*wR*(*F*^2^) = 0.143	H-atom parameters constrained
*S* = 1.03	*w* = 1/[σ^2^(*F*_o_^2^) + (0.0633*P*)^2^ + 0.1946*P*] where *P* = (*F*_o_^2^ + 2*F*_c_^2^)/3
3684 reflections	(Δ/σ)_max_ < 0.001
217 parameters	Δρ_max_ = 0.38 e Å^−^^3^
0 restraints	Δρ_min_ = −0.19 e Å^−^^3^

### Special details

**Table:**

Geometry. Bond distances, angles *etc*. have been calculated using the rounded fractional coordinates. All su's are estimated from the variances of the (full) variance-covariance matrix. The cell e.s.d.'s are taken into account in the estimation of distances, angles and torsion angles
Refinement. Refinement of *F*^2^ against ALL reflections. The weighted *R*-factor *wR* and goodness of fit *S* are based on *F*^2^, conventional *R*-factors *R* are based on *F*, with *F* set to zero for negative *F*^2^. The threshold expression of *F*^2^ > σ(*F*^2^) is used only for calculating *R*-factors(gt) *etc*. and is not relevant to the choice of reflections for refinement. *R*-factors based on *F*^2^ are statistically about twice as large as those based on *F*, and *R*- factors based on ALL data will be even larger.

### Fractional atomic coordinates and isotropic or equivalent isotropic displacement parameters (Å^2^)

**Table:**

	*x*	*y*	*z*	*U*_iso_*/*U*_eq_	
O1	0.43825 (10)	0.23381 (9)	0.0566 (2)	0.0651 (5)	
O2	0.57029 (10)	0.25445 (10)	−0.0177 (2)	0.0762 (6)	
O3	0.81423 (10)	0.03761 (12)	0.1565 (2)	0.0771 (6)	
O4	0.78682 (11)	−0.10369 (11)	0.2563 (2)	0.0819 (7)	
N1	0.34097 (9)	0.00794 (9)	0.26919 (19)	0.0434 (5)	
N2	0.39945 (9)	0.07682 (9)	0.2205 (2)	0.0442 (5)	
N3	0.51727 (11)	0.20674 (10)	0.0563 (2)	0.0491 (5)	
N4	0.76344 (11)	−0.02141 (13)	0.2090 (2)	0.0567 (6)	
C1	0.19535 (12)	−0.03794 (12)	0.3219 (2)	0.0458 (6)	
C2	0.22711 (13)	−0.12656 (13)	0.3948 (3)	0.0565 (7)	
C3	0.16793 (15)	−0.19545 (15)	0.4405 (3)	0.0679 (8)	
C4	0.07590 (15)	−0.17652 (17)	0.4138 (3)	0.0738 (9)	
C5	0.04314 (14)	−0.08992 (18)	0.3402 (3)	0.0733 (9)	
C6	0.10145 (13)	−0.01864 (14)	0.2944 (3)	0.0572 (7)	
C7	0.06687 (14)	0.07705 (17)	0.2168 (4)	0.0770 (9)	
C8	0.13139 (15)	0.15586 (16)	0.2858 (4)	0.0773 (9)	
C9	0.22538 (13)	0.13744 (13)	0.2358 (3)	0.0583 (7)	
C10	0.25900 (12)	0.03616 (12)	0.2750 (2)	0.0448 (6)	
C11	0.48721 (11)	0.05420 (10)	0.2161 (2)	0.0361 (5)	
C12	0.54739 (11)	0.11646 (10)	0.1415 (2)	0.0380 (5)	
C13	0.63792 (11)	0.09211 (11)	0.1416 (2)	0.0415 (5)	
C14	0.66902 (11)	0.00529 (12)	0.2126 (2)	0.0421 (5)	
C15	0.61171 (12)	−0.05889 (12)	0.2843 (2)	0.0441 (6)	
C16	0.52342 (12)	−0.03497 (11)	0.2866 (2)	0.0407 (5)	
H2	0.28932	−0.13939	0.41286	0.0677*	
H2A	0.37998	0.13420	0.19292	0.0530*	
H3	0.18999	−0.25435	0.48905	0.0815*	
H4	0.03567	−0.22256	0.44564	0.0885*	
H5	−0.01936	−0.07871	0.32068	0.0880*	
H7A	0.05919	0.07480	0.08332	0.0924*	
H7B	0.00786	0.09011	0.25190	0.0924*	
H8A	0.13617	0.16063	0.41865	0.0928*	
H8B	0.10803	0.21668	0.23270	0.0928*	
H9A	0.22348	0.15075	0.10567	0.0699*	
H9B	0.26846	0.18190	0.30438	0.0699*	
H13	0.67680	0.13436	0.09391	0.0499*	
H15	0.63399	−0.11814	0.33062	0.0529*	
H16	0.48587	−0.07817	0.33571	0.0489*	

### Atomic displacement parameters (Å^2^)

**Table:**

	*U*^11^	*U*^22^	*U*^33^	*U*^12^	*U*^13^	*U*^23^
O1	0.0644 (10)	0.0433 (7)	0.0887 (11)	0.0134 (7)	0.0152 (8)	0.0104 (7)
O2	0.0709 (10)	0.0599 (9)	0.0990 (12)	−0.0085 (7)	0.0170 (9)	0.0319 (8)
O3	0.0478 (9)	0.0901 (11)	0.0970 (12)	−0.0040 (8)	0.0226 (8)	0.0040 (9)
O4	0.0619 (10)	0.0738 (10)	0.1095 (14)	0.0256 (8)	0.0125 (9)	0.0107 (9)
N1	0.0395 (8)	0.0434 (8)	0.0471 (8)	−0.0049 (6)	0.0066 (6)	−0.0047 (6)
N2	0.0424 (8)	0.0372 (7)	0.0531 (9)	0.0006 (6)	0.0079 (7)	−0.0007 (6)
N3	0.0559 (10)	0.0375 (7)	0.0533 (9)	−0.0033 (7)	0.0069 (7)	0.0010 (6)
N4	0.0457 (10)	0.0653 (10)	0.0588 (10)	0.0039 (8)	0.0071 (8)	−0.0069 (8)
C1	0.0393 (10)	0.0505 (10)	0.0474 (10)	−0.0031 (8)	0.0060 (8)	−0.0076 (8)
C2	0.0457 (11)	0.0504 (10)	0.0745 (14)	−0.0018 (9)	0.0132 (10)	−0.0057 (9)
C3	0.0658 (14)	0.0512 (11)	0.0893 (17)	−0.0065 (10)	0.0202 (12)	−0.0017 (11)
C4	0.0581 (14)	0.0675 (14)	0.0990 (18)	−0.0195 (11)	0.0224 (12)	−0.0061 (12)
C5	0.0400 (12)	0.0878 (16)	0.0923 (18)	−0.0107 (11)	0.0112 (11)	−0.0032 (13)
C6	0.0407 (11)	0.0678 (12)	0.0619 (13)	0.0001 (9)	0.0050 (9)	−0.0042 (10)
C7	0.0446 (12)	0.0881 (16)	0.0959 (19)	0.0085 (12)	0.0039 (12)	0.0159 (13)
C8	0.0580 (14)	0.0692 (14)	0.1048 (19)	0.0188 (12)	0.0134 (12)	0.0113 (13)
C9	0.0490 (12)	0.0518 (11)	0.0742 (14)	0.0067 (9)	0.0103 (10)	0.0042 (9)
C10	0.0406 (10)	0.0487 (9)	0.0440 (10)	0.0008 (8)	0.0039 (8)	−0.0066 (8)
C11	0.0393 (9)	0.0349 (8)	0.0335 (8)	−0.0029 (7)	0.0036 (7)	−0.0074 (6)
C12	0.0443 (10)	0.0312 (7)	0.0372 (9)	−0.0027 (7)	0.0026 (7)	−0.0039 (6)
C13	0.0422 (10)	0.0414 (9)	0.0413 (9)	−0.0092 (7)	0.0075 (7)	−0.0052 (7)
C14	0.0374 (9)	0.0454 (9)	0.0426 (10)	−0.0001 (7)	0.0040 (7)	−0.0074 (7)
C15	0.0485 (11)	0.0374 (8)	0.0441 (10)	0.0009 (8)	0.0005 (8)	−0.0004 (7)
C16	0.0444 (10)	0.0365 (8)	0.0406 (9)	−0.0041 (7)	0.0047 (7)	−0.0008 (7)

### Geometric parameters (Å, °)

**Table:**

O1—N3	1.233 (2)	C9—C10	1.503 (2)
O2—N3	1.221 (2)	C11—C16	1.414 (2)
O3—N4	1.217 (2)	C11—C12	1.415 (2)
O4—N4	1.227 (2)	C12—C13	1.387 (2)
N1—N2	1.3769 (19)	C13—C14	1.363 (2)
N1—C10	1.287 (2)	C14—C15	1.393 (2)
N2—C11	1.347 (2)	C15—C16	1.356 (3)
N3—C12	1.439 (2)	C2—H2	0.9300
N4—C14	1.456 (2)	C3—H3	0.9300
N2—H2A	0.8600	C4—H4	0.9300
C1—C2	1.393 (2)	C5—H5	0.9300
C1—C6	1.404 (3)	C7—H7A	0.9700
C1—C10	1.475 (2)	C7—H7B	0.9700
C2—C3	1.376 (3)	C8—H8A	0.9700
C3—C4	1.376 (3)	C8—H8B	0.9700
C4—C5	1.374 (3)	C9—H9A	0.9700
C5—C6	1.391 (3)	C9—H9B	0.9700
C6—C7	1.502 (3)	C13—H13	0.9300
C7—C8	1.489 (3)	C15—H15	0.9300
C8—C9	1.522 (3)	C16—H16	0.9300
			
N2—N1—C10	115.97 (13)	N4—C14—C13	119.11 (15)
N1—N2—C11	119.91 (12)	C13—C14—C15	121.27 (16)
O1—N3—O2	121.22 (15)	C14—C15—C16	120.00 (15)
O1—N3—C12	119.61 (14)	C11—C16—C15	121.45 (15)
O2—N3—C12	119.16 (15)	C1—C2—H2	119.00
O3—N4—O4	123.60 (17)	C3—C2—H2	119.00
O3—N4—C14	118.89 (16)	C2—C3—H3	120.00
O4—N4—C14	117.49 (16)	C4—C3—H3	120.00
C11—N2—H2A	120.00	C3—C4—H4	120.00
N1—N2—H2A	120.00	C5—C4—H4	120.00
C2—C1—C10	120.96 (16)	C4—C5—H5	119.00
C6—C1—C10	119.59 (15)	C6—C5—H5	119.00
C2—C1—C6	119.45 (17)	C6—C7—H7A	109.00
C1—C2—C3	121.07 (18)	C6—C7—H7B	109.00
C2—C3—C4	119.5 (2)	C8—C7—H7A	109.00
C3—C4—C5	120.3 (2)	C8—C7—H7B	109.00
C4—C5—C6	121.4 (2)	H7A—C7—H7B	108.00
C1—C6—C7	119.67 (17)	C7—C8—H8A	109.00
C5—C6—C7	122.09 (18)	C7—C8—H8B	109.00
C1—C6—C5	118.24 (18)	C9—C8—H8A	109.00
C6—C7—C8	110.9 (2)	C9—C8—H8B	109.00
C7—C8—C9	111.14 (19)	H8A—C8—H8B	108.00
C8—C9—C10	113.60 (16)	C8—C9—H9A	109.00
C1—C10—C9	119.35 (16)	C8—C9—H9B	109.00
N1—C10—C9	124.42 (16)	C10—C9—H9A	109.00
N1—C10—C1	116.23 (15)	C10—C9—H9B	109.00
N2—C11—C16	120.55 (14)	H9A—C9—H9B	108.00
N2—C11—C12	122.79 (13)	C12—C13—H13	120.00
C12—C11—C16	116.66 (15)	C14—C13—H13	121.00
N3—C12—C13	116.51 (14)	C14—C15—H15	120.00
N3—C12—C11	121.84 (15)	C16—C15—H15	120.00
C11—C12—C13	121.62 (13)	C11—C16—H16	119.00
C12—C13—C14	118.99 (14)	C15—C16—H16	119.00
N4—C14—C15	119.61 (15)		
			
C10—N1—N2—C11	178.11 (14)	C2—C3—C4—C5	0.7 (3)
N2—N1—C10—C9	−1.1 (2)	C3—C4—C5—C6	−1.3 (3)
N2—N1—C10—C1	178.32 (13)	C4—C5—C6—C7	−179.0 (2)
N1—N2—C11—C12	170.32 (14)	C4—C5—C6—C1	1.2 (3)
N1—N2—C11—C16	−9.3 (2)	C1—C6—C7—C8	−36.0 (3)
O1—N3—C12—C11	4.1 (2)	C5—C6—C7—C8	144.2 (2)
O2—N3—C12—C11	−174.74 (15)	C6—C7—C8—C9	58.4 (3)
O2—N3—C12—C13	3.3 (2)	C7—C8—C9—C10	−46.4 (3)
O1—N3—C12—C13	−177.87 (14)	C8—C9—C10—N1	−169.50 (18)
O4—N4—C14—C15	5.9 (2)	C8—C9—C10—C1	11.2 (2)
O4—N4—C14—C13	−172.68 (15)	N2—C11—C16—C15	−179.66 (14)
O3—N4—C14—C15	−175.53 (15)	C16—C11—C12—C13	−1.5 (2)
O3—N4—C14—C13	5.9 (2)	C16—C11—C12—N3	176.45 (13)
C10—C1—C6—C7	0.3 (3)	C12—C11—C16—C15	0.7 (2)
C2—C1—C6—C7	179.7 (2)	N2—C11—C12—N3	−3.2 (2)
C10—C1—C6—C5	−179.91 (17)	N2—C11—C12—C13	178.86 (14)
C2—C1—C10—C9	−166.92 (17)	C11—C12—C13—C14	1.1 (2)
C6—C1—C10—C9	12.5 (2)	N3—C12—C13—C14	−176.90 (14)
C2—C1—C10—N1	13.7 (2)	C12—C13—C14—N4	178.59 (14)
C2—C1—C6—C5	−0.5 (3)	C12—C13—C14—C15	0.1 (2)
C6—C1—C2—C3	−0.1 (3)	N4—C14—C15—C16	−179.38 (14)
C10—C1—C2—C3	179.31 (18)	C13—C14—C15—C16	−0.9 (2)
C6—C1—C10—N1	−166.93 (16)	C14—C15—C16—C11	0.5 (2)
C1—C2—C3—C4	0.0 (3)		

### Hydrogen-bond geometry (Å, °)

**Table:**

*D*—H···*A*	*D*—H	H···*A*	*D*···*A*	*D*—H···*A*
N2—H2A···O1	0.86	1.99	2.5976 (19)	127
C2—H2···O2^i^	0.93	2.57	3.426 (2)	153
C15—H15···O1^i^	0.93	2.52	3.235 (2)	134

Symmetry codes: (i) −*x*+1, *y*−1/2, −*z*+1/2.
